# Monitoring the Landscape Pattern and Characteristics of Non-Point Source Pollution in a Mountainous River Basin

**DOI:** 10.3390/ijerph182111032

**Published:** 2021-10-20

**Authors:** Yuepeng Liu, Chuanfeng Yang, Xinyang Yu, Mengwen Wang, Wei Qi

**Affiliations:** 1 College of Resources and Environment, Shandong Agricultural University, Tai’an 271018, China; seashells7@126.com (Y.L.); y13861510723@163.com (C.Y.); yuxinyang19860915@163.com (X.Y.); tsuls_wmw@163.com (M.W.); 2 Natural Resources and Planning Bureau of Yixing County, Wuxi 214200, China; 3 School of Economics and Management, Taishan University, Tai’an 271000, China

**Keywords:** non-point source pollution, “source-sink” theory, location weighted landscape contrast index (*LWLCI*), landscape patterns

## Abstract

This study aimed to assess the relationship between the landscape patterns and non-point source (NPS) pollution distribution in Qixia County, China. The sub-basin classification was conducted based on a digital elevation model and Landsat8 satellite images. Water samples were collected from each sub-basin, andtheir water quality during the wet and dry seasons was estimated. The correlation between the landscape indices and water pollution indicators was determined by Pearson analysis. The location-weighted landscape contrast index (*LWLCI*) was calculated based on the “source-sink” theory. Qixia was further divided into five sections based on the *LWLCI* score to illustrate the potential risk of NPS pollution. The results showed that the water quality in Qixia County was generally good. Cultivated land, orchards, construction areas, and unused land were positively correlated with the water pollution index and weredesignated as the “source” landscape categories, while forests, grasslands, and water bodies, which were negatively correlated with water pollution, were the “sink” landscapes; the *LWCI* was high in 36.94% of the study area. In these areas, measures such as increasing vegetation buffer zones are necessary to decrease the sediment and nutrient loads carried by precipitation.

## 1. Introduction

Depending on the source, water pollution can be categorized as point and non-point source pollution [1]. Point source pollution originates from a single source, while non-point source (NPS) pollution is caused by various undefined pollutants, such as soil sediments, livestock fecal sewage, and solid waste [2]. For example, the movement of rainwater or irrigation water can carry pollutants, such as fertilizers, herbicides, and pesticides, into rivers, lakes, reservoirs, and underground waters [3]. With the rapid economic development and high population growth in recent years, NPS pollution has become the main cause of water pollution [4], which mostly carries large amounts of N and P (NPS-N and NPS-P, respectively). The increase in NPS water pollution has caused serious hydrological and environmental problems worldwide and become a major challenge in environmental protection [5,6]. Although NPS has attracted much attention for its effects on human and ecosystem health, it is almost impossible to regulate due to the uncertainty of its sources and the complexity of its transmission process [7]. Accurate monitoring of the changing patterns of NPS pollution is essential for combating, controlling, and reducing water pollution. Studies on NPS pollution have mainly focused on source identification, characterization, hazard assessment, mechanism discussion, and the factors influencing NPS pollution, and mainly involved field surveys, monitoring, and simulation [8]. Research shows that NPS pollution is the main cause of surface water pollution, with agricultural NPS pollution making the greatest contribution [9,10,11]. Landscape patterns can determine the amount of pollutants reaching water bodies through different ecological and hydrological factors [4]. Therefore, landscape pattern change has long been considered as a major factor influencing the extent and transmission of NPS pollution [12]. Numerous researchers have explored this topic through the application of hydrological models and geographic information systems (GIS). Ouyang et al. used the SWAT model to illustrate the diffuse pollution dynamics and their response to land development in the Yangtze River [13]. The Slurp model was utilized by Jain et al. (1998) and Chen (2013) to analyze the load estimation and source apportionment of NPS pollution in the Satluj catchment and Jinjiang River, respectively [14,15]. The sparrow model was incorporated in a study of fluvial suspended sediment transport in the non-tidal streams of the Chesapeake Bay water body and its vicinity [16]. Such modelscan both quantify the amount of NPS pollutants produced and simulate the attenuation and transformation of these pollutants during transport. Although they have been extensively used, they require massive data input for simulation and a large amount of time for manipulation [10].

China has pledged to fight water pollution caused by NPSs. The Action Plan for Prevention and Control of Water Pollution (2015) published by the Ministry of Ecology and Environment of the People’s Republic of China includes specific chapters related to combating NPS pollution [17,18]. However, more technologies for NPS pollution control and monitoring in accordance with local landscape features are yet to be developed [19].

In 2003, Chen proposed that the “source-sink” theory should be used to better illustrate the correlation between landscape patterns and water quality [20,21]. The location-weighted landscape contrast index (*LWLCI*) was also proposed by Chen based on the “source-sink” theory [20]. Chen suggested using the Lorentz curve to determine the spatial distribution pattern of “source” and “sink”. The Lorentz curve was originally used to describe the distribution of wealth among different groups of people and demonstrates the accumulation of one factor in an increasing process, as well as other related variables. This theory has been widely used to study many ecological processes, such as NPS pollution, soil erosion, and carbon loss. Jiang et al. analyzed NPS pollution in the Jiulongjiang Estuary by constructing a grid landscape contrast index based on the spatial load contrast index [2]. Wu studied the relationship between soil carbon loss and landscape indicators based on the *LWLCI* [22]. Chen et al. used the *LWLCI* to study the interactions between urban landscape patterns and land surface temperatures [8]. Furthermore, Wang examined the effects of land cover change on the soil erosion process [23], and Olivera used the Land-Cover Pollution Index (LCPI) to evaluate the relationship between land use and cover change (LUCC) and water quality [6]. In most of these studies, only *LWLCI* was calculated based on the land configuration of the research area, and few studies have determined the relationship between the *LWLCI* and water quality indicators.

With technological advancement and economic development, human activities are increasingly affecting the environment. NPS pollution is more likely to be influenced by watershed landscape patterns than point source pollution. This study aimed to identify the key areas for the prevention and control of NPS pollution in the study area based on the “Source and Sink” theory. Mitigation measures based on our results would aid in managing NPS pollution in the watershed, creating a spatial pattern conducive to regional human health, and promoting the sustainable development of the region. The objective of this study was to determine the relationship between water quality and the landscape and provide a scientific basis for authorities to implement measures to ensure the health of residents and local sustainability. To better illustrate the relationship between landscape patterns and NPS pollution, Pearson correlation analysis was conducted between the landscape and water quality indicators, and the *LWLCI* was calculated to illustrate the quantitative relationship between NPS and landscape configuration. Future mitigation measures were also proposed based on a literature review and expert opinions.

## 2. Materials and Methods

### 2.1. Study Region

**Figure 1:**
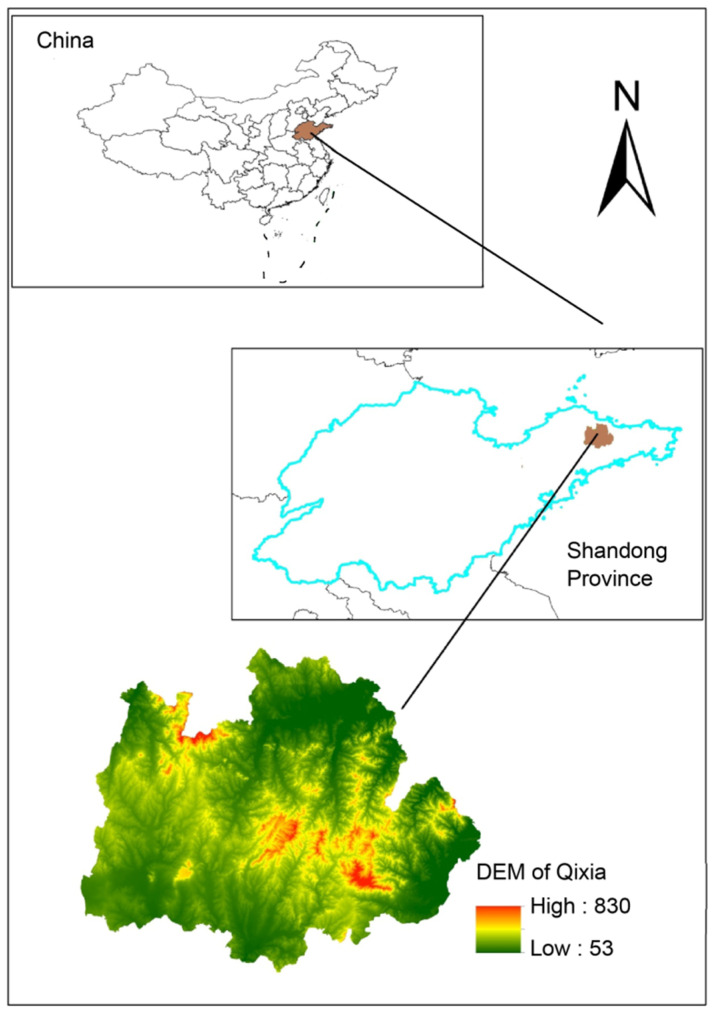
Above to below: location of Shandong Province in China, location of Qixia in Shandong Province, and Digital Elevation Model (DEM) of Qixia.

Qixia County (120°33′–121°15′ E, 37°05′–37°32′ N) was selected as the study region, which is characterized by mountainous and hilly terrain with a total area of 2017 km^2^. The altitude in the study region ranges from 53 to 830 m (Figure 1). The main soil types are mountain yellow-brown earth, alluvial soil, and aquic brown soil [24]. Qixia receives 743.1 mm of annual precipitation, four distinct seasons, large amount of sunshine, and a moderate amount of rainfall. The mean annual temperature of Qixia is 11.3 °C, with a frost-free period of 207 d and annual actual sunshine duration of 2760 h. It experiences a warm, temperate monsoon climate [24]. Qixia consists of six main tributaries with two dams, with a total length of approximately 184 km. The water area is approximately 1685 km^2^. According to the Qixia Yearbook 2018, the average daily water usage per person is approximately 117.82 L, and the annual water usage of the whole county is 4.19 million m^3^ [25].

Qixia County is located in a hilly, mountainous area. The soils of Qixia can be divided into two main categories: brown loam and alluvial soil. Brown loam, a significantly alkaline soil, covers approximately 70% of the city’s arable land area, accounting for 88.6% of the total sown area. Alluvial soils are mostly found in the alluvial plains of river valleys [25]. The intrusive rocks of the valleys and plains in Qixia are mainly fine and medium-grained diorite granites from the Early Yuan Dynasty Luliang [26]. However, groundwater is shallow and vulnerable to environmental change. Therefore, unified management and protection should be strengthened to avoid groundwater contamination.

Based on data from the website of the Qixia Government, Longmenkou Reservoir provides the city with drinking water. The main pollutants affecting the water quality are chemical oxygen demand (COD) and total nitrogen, and the overall pH is weakly alkaline [27]. Water quality data are provided in Table 1.

As of 2017, Qixia has a total population of 485,400, birth rate of 0.809%, mortality rate of 1.044%, and natural population growth rate of −0.234% [25]. The death rates for men and women were 961.74 per 100,000 and 795.40 per 100,000 in 2017, respectively. In 2014, a total of 1876 confirmed cases of tumors were reported in Qixia, with an incidence rate of 3022.19 per 100,000; for men, the total number of cases and incidence rate were 1123 and 3504.34 per 100,000, respectively, while the number of cases and incidence rate for women were 753 and 2477.80 per 100,000, respectively. The top 10 malignant tumors in order of incidence rate were caused by stomach, lung, liver, colorectal, breast, bladder, uterine body, esophageal, and pancreatic cancer, accounting for a total of 15,911 cases and 84.81% of the total [28]. Qixia County is part of Yantai City. According to Wang, Yantai has the highest incidence and mortality rates of liver cancer among all cities in Shandong Province [29].

Water quality is correlated with the incidence of malignant tumors and cancer [30]. A previous study reported that COD and biological oxygen demand (BOD) in drinking water are closely, positively related to the liver cancer death rate in southern China [31]. Forman et al. found that drinking water containing high levels of nitrates could result in the enlargement of the thyroid gland, and increased incidence of 15 types of cancer and two types of birth defects in humans [32]. Several studies have also demonstrated that excessive (NH_4_)_2_SO_4_ and NH_4_NO_3_ in drinking water could exert detrimental effects on human reproduction, causing abnormal decreases in body weight [33].

### 2.2. Spatial Data

**Figure 2:**
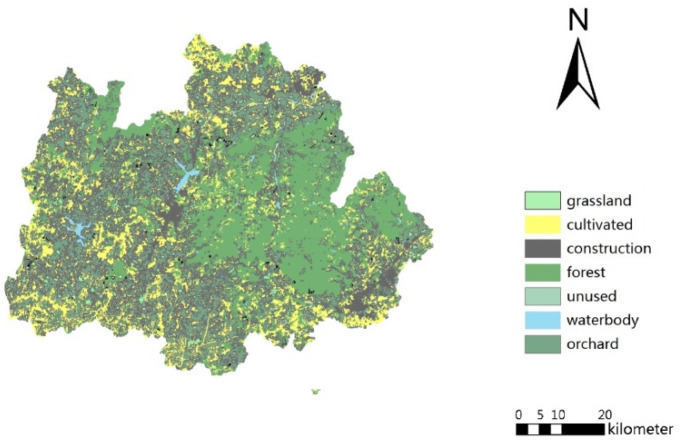
Land-use classification of Qixia.

A Landsat8 remote sensing image of Qixia County with a resolution of 30 m was acquired from the China Geospatial Data Cloud website. Geometric precision correction, spectral processing, and atmospheric correction of the acquired image were conducted. Based on the Chinese Land Use Classification Standard, the landscape in the study region was classified into seven types: cultivated land, forests, orchards, construction land, water bodies, grasslands, and unused land (Figure 2). The classification was conducted based on visual interpretation to ensure classification accuracy. A digital elevation model (DEM) with a 30-m resolution was acquired from the National Fundamental Science Data Sharing Platform. Annual precipitation and fertilizer application data were obtained from the Chinese meteorological data website and a local yearbook, respectively. Approximately 126,501 t of fertilizer was applied over 875 km^2^ of total sown land in Qixia2017. Fertilizer usage in China, Shandong Province, and Qixia County is compared in Table 2. The usage data for Shandong and China in 2018 were acquired from the Chinese National Bureau of Statistics inquiry website [34].

Both the average fertilizer and pesticide usage in Shandong slightly exceeded the national average, while the usage in Qixia was over three times higher than the national average. The large area of apple orchards in Qixia could be an explanation for this, as the average fertilizer usage for orchards in China during 2014 was approximately 93.19 t/km^2^ [34]. Shandong applied 1,306,700, 421,300, and 356,400 t of N-fertilizer, P-fertilizer, and K-fertilizer during 2018, accounting for 31%, 10%, and 8% of the total fertilizer usage, respectively. Based on these percentages, we calculated that 39,324.11, 12,678.69, and 10,725 t of N-fertilizer, P-fertilizer, and K-fertilizer was applied in Qixia during 2017, respectively. As the fertilizer utilization rate in China is 30–50% [35], approximately 23,594.46, 7607.2, and 6435.34 t of N-fertilizer, P-fertilizer, and K-fertilizer remained in the soil and river systems of Qixia, posing a great hazard to local public health.

### 2.3. Field Survey Data

**Figure 3:**
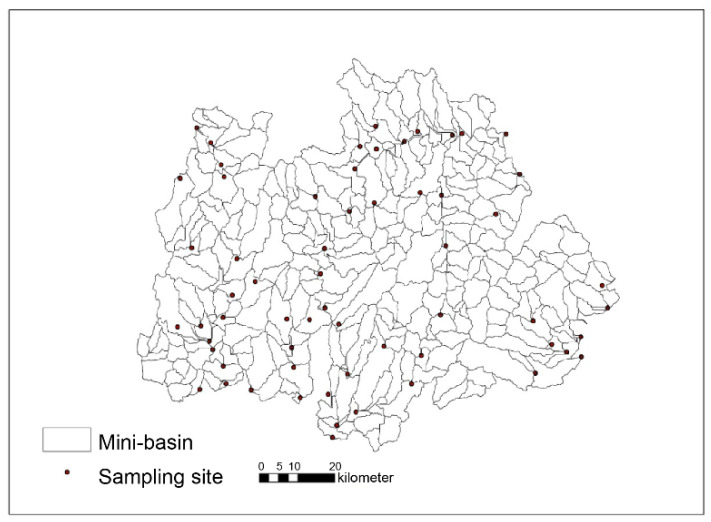
The sixty-one sampling sites selected in this study.

Water samples were collected from 61 sites within the study area to better understand the correlation between water quality and landscape distribution (Figure 3). These 61 sites were selected to represent various land-use types and morphologies. According to the Qixia Yearbook, the average precipitation in May is only 7.7 mm, while precipitation in July and August altogether (490mm) accounts for more than 60% of Qixia annual total precipitation [25]. Samples (1000 mL) were collected from each site weekly during May and early September 2016 to reflect the water quality in the dry and wet seasons. The samples were immediately sealed in an incubator after collection and processed for 24 h. The water quality indicators analyzed in this study included the total N (TN), ammonium N (NH_4_^+^-N), total P (TP), and chemical oxygen demand (COD). The NH_4_^+^-N, and TN, COD, and TP contents were determined using a continuous-flow injection, and following the potassium persulfate oxidation spectrophotometric, potassium chlorinate, and ammonium molybdate spectrophotometric methods, respectively. The tests were repeated thrice for each sample, and the final results for each indicator were recorded after averaging the experimental results.

The water quality was determined following the comprehensive pollution index method, which is based on the evaluation of the functional areas of a water environment. The index was constructed via the following steps: (1) measuring individual water quality indicators and determining corresponding water quality standards in each water environment category; (2) applying arithmetic average, multiplication, weighted average, and other mathematical methods to the individual water quality indicators and generating a comprehensive pollution index that reflects water conditions and quality [36]. The weighted average was used to calculate the composite pollution index and quantify the water quality indicators of each sub-basin using the Formula (1) [36]:

(1) $$P=\frac{1}{n}\sum_{i=1}^{n}P_{i}=\frac{1}{n}\sum_{i=1}^{n}\frac{c_{i}}{s_{i}}$$

where *P* is the comprehensive pollution index, *n* is the number of river water quality indicators involved in the evaluation, *P_i_* is the pollution index of pollutant category *i*, *C_i_* is the average value of pollutant category *i* (mg/L), and *S_i_* is the evaluation standard of pollutant category *i* (mg/L). The higher the value of *P*, the more severe the pollution condition of the water body [37].

The independent-samples *t*-test is the analysis of whether there is a significant difference in the sample data and is conducted by testing the means of two samples from two independent subjects [22]. In this study, by calculating the comprehensive pollution index of water quality in the dry and tributary streams of different small water bodies, an independent-samples *t*-test was conducted using river water quality indicators to further elucidate the differences in water quality between the dry and tributary streams of Qixia City.

### 2.4. Sub-Basin Partitioning

This study used the Hydrology module in ArcGIS 10.2 (ESRI, Redlands, CA, USA) to extract the water body boundaries by combining the DEM of the study area to extract the flow direction of the surface water runoff model, calculate the sink flow accumulation, and generate the river network to partition the water body in the study area.

### 2.5. Landscape Pattern Analysis

Landscape metrics are a quantitative characterization of landscape pattern characteristics. Landscape spatial pattern variables based on landscape indices are commonly used in the literature [38]. Using Fragstats 4.2, five landscape metrics, i.e., patch density (PD), largest patch index (LPI), landscape shape index (LSI), interspersion and juxtaposition index, contagion index (CONTAG), and Shannon’s diversity index (SHDI), were calculated to illustrate the heterogeneity of the study area. The correlation between landscape patterns and water quality was explored using Pearson analysis in SPSS 19.0.

### 2.6. “Source-Sink” and LWLCI

The “source-sink” theory posits that a certain landscape type can be categorized as either a “source” or “sink” based on its corresponding “source” or “sink” function in ecological processes. In this study, for NPS pollution, the landscape types that can provide or increase the volume of pollutants, such as farmland, construction fields, and orchards, were categorized as “source” types, while those that can absorb or alleviate the pollution load, namely forests, water, and grasslands, were categorized as “sink” types. This categorization was verified by the Pearson analysis results [39]. The total area and percentage of “source” and “sink” areas are listed in Table 3.

**Figure 4:**
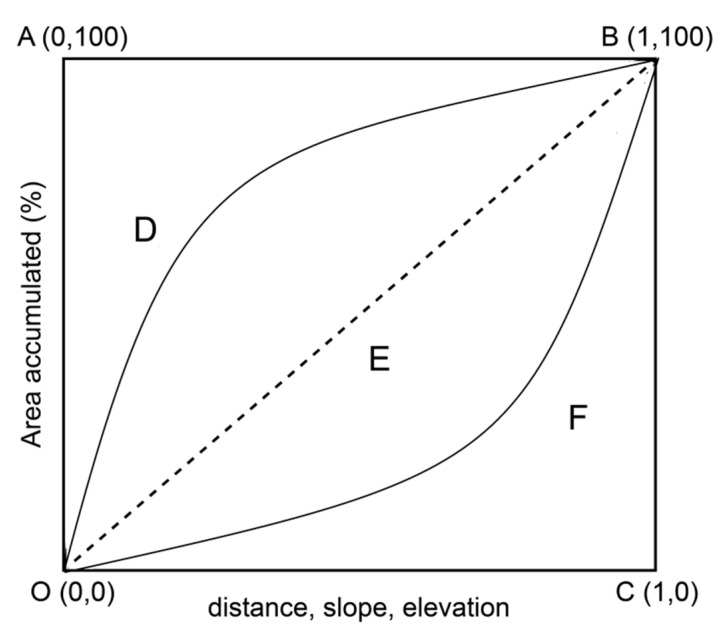
Distribution of “source” and “sink” landscape types. Adapted from [21].

The Lorentz curve diagram is shown in Figure 4 [21]. The O-E-B line represents the absolute even distribution curve of landscape types in the water body; if the “source” and “sink” landscapes are evenly distributed in the basin, the OEB distribution curve would appear. In this case, the effects on NPS pollution from the “source” and “sink” landscapes were the same, and the landscape pattern was theoretically in equilibrium. If the “source-sink” landscape is unevenly distributed in space, as shown by the O-D-B and O-F-B lines (assuming that O-D-B and O-F-B represent the “source” and “sink” landscape types, respectively), their contribution to the monitoring points at the river basin exit could be determined by the area accumulation curve of each landscape type and the area of the irregular triangle composed of the O-C and C-B straight lines. Thus, the *LWLI*′ can be represented as Formula (2) [20,21]:

(2) $${LWLI}^{\prime }=\sum_{i=1}^{m}A_{sourcei}\times W_{i}\times AP_{i}÷[\sum_{i=1}^{m}A_{sourcei}\times W_{i}\times AP_{i}+\sum_{j=1}^{n}A_{\sin kj}\times W_{j}\times AP_{j}]$$

Considering that the relative height, slope, and distance between landscape elements and surface water are the major influencing factors, the *LWLCI* for these landscape elements can be calculated as Formula (3) [20,21]:

(3) $$LWLCI=LWLCI_{distance}\times LWLCI_{elevation}÷LWLCI_{slope}\;$$

where *LWLCI* represents the comprehensive landscape spatial load comparison index, sink *j* represents the area of an irregular triangle composed of “source” and “sink” landscape types, m and n represent the number of “source” and “sink” landscapes, respectively, *W_i_* and *W_j_* represent the weight of various “source” and “sink” landscapes, and *AP_i_* and *AP_j_* represent the percentage of the area of various “source” and “sink” landscapes in each small water body, respectively.

The weight allocation process is mainly based on empirical studies and expert opinions. Wang determined that the weight of a given landscape is related to its significance in the soil erosion process [11]. In light of this, in this study, farmland was assigned a weight value of 0.8 as it is a key pollution source, whereas orchards and construction land were assigned a weight value of 0.6 due to their potential contributions to pollution. The weight values of “sink” landscapes were determined by comparing their effects in mitigating pollution (Table 4). It is important to note that the *LWLCI* of NPS pollution zoning is a comprehensive index based on elevation, slope, distance, and landscape type, and can better reflect all types of water bodies.

## 3. Results

### 3.1. Water Quality and Sub-Basin Partitioning

The test results showed that 94.73% of the cross-section water quality in Qixia at least met the Class-III standard at a minimum, according to the Environmental Quality Standards for Surface Water established by the Ministry of Ecology and Environment of the People’s Republic of China [40] (Table 5).

**Figure 5:**
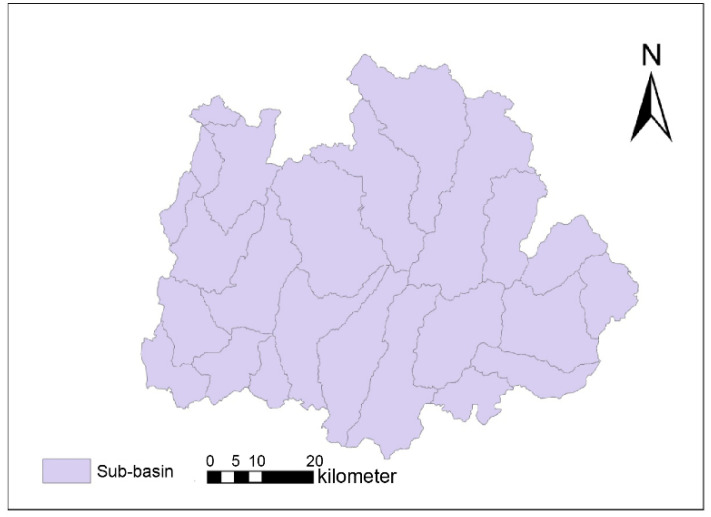
Sub-basin partitioning of Qixia.

Combining the distribution characteristics of water bodies and river paths in the satellite image data, Qixia was initially divided into 300 mini-basins. After merging and manually modifying the boundaries of the water bodies in Qixia, they were finally divided into 21 sub-basins, as shown in Figure 5.

### 3.2. Correlation between Land Use and Water Quality Factors

**Figure 6:**
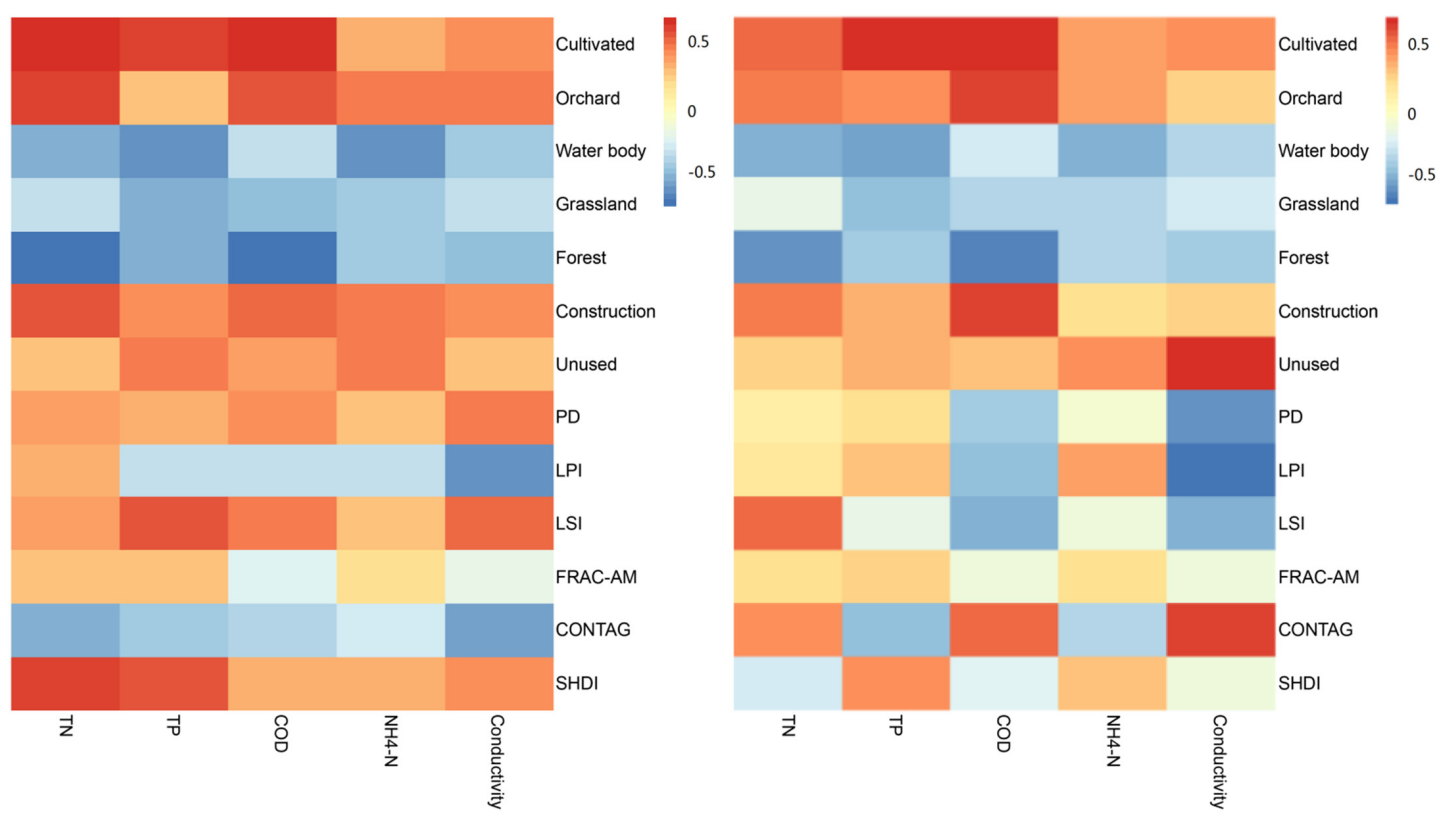
Heat maps of the correlation between land use and water quality factors.

Spatial correlation analysis of the landscape spatial load contrast index and its correlation with the spatial distribution of the water body water quality index involves a collection of a series of spatial data analysis methods and techniques. Our Pearson analysis results showed that the proportion of land use areas and each water quality indicator were correlated (Figure 6). The areas characterized as cultivated land, orchards, construction land, and unused land were positively correlated with water quality indicators and were the main “source” types of the studied landscape. Forests, grasslands, and water areas had a negative correlation with water quality. The positive correlation of cultivated land and orchards with water quality was greater than that of construction land and water quality. The negative correlation between forests and water quality indicators was greater than that between grasslands and water quality indicators.

### 3.3. LWLCI Division

**Figure 7:**
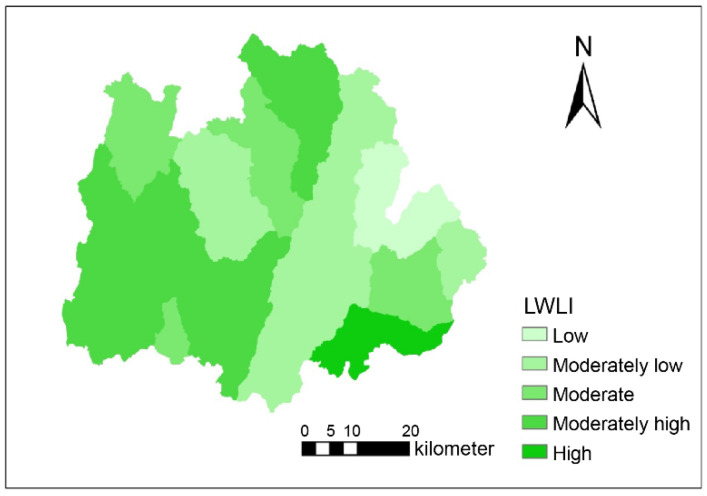
Location-weighted landscape contrast index (*LWLCI*) zoning of Qixia.

To better demonstrate the distribution of the *LWLCI* in Qixia, the “source-sink” landscape spatial index map of Qixia County was created using SPSS 19.0(IBM, Armonk, NY, USA), Excel 2010 (Microsoft, Redmond, WA, USA), and ArcGIS 10.2 (ESRI, Redlands, CA, USA) (Figure 7). Overall, the landscape spatial index was higher in hilly, comprehensive grain-fruit utilization areas in southwestern Qixia, southern Taocun Town, and most of Zangjiazhuang Town, and lower in the low mountain forest protection area in eastern Qixia. The proportion of areas with moderately high *LWLCI* was the highest (37.47%), followed by areas with moderately low *LWLCI*, whereas the proportion of areas with high *LWLCI* was the lowest (4%). The global Moran index of *LWLCI* was 0.701 (*p* < 0.01) (Table 6). The correlations between *LWLCI* and water quality indicators during the wet and dry seasons are shown in Table 7.

## 4. Discussion

Our results confirmed that landscape configuration can significantly affect the load and distribution of NPS pollution. Cultivated land, orchards, and construction areas can enhance the transmission of pollutants, while forests, grasslands, and water bodies have the opposite effect. Our differentiation of “source” and “sink” landscapes was mainly based on experience and experimental results. It should be noted that a certain land-use type can shift from a “source” or “sink” or vice versa in the same ecological process depending on the research subjects. For example, in this study, forests acted as “sink” landscapes in evaluating NPS-T or NPS-P, but they may become “source” landscapes in terms of organic matter NPS pollution. This is because the organic matter in forest soil can be transmitted into rivers under heavy rainfall [41]. In this study, organic matter pollution was not considered in this study. Scientifically categorizing “source” and “sink” landscapes and how “source” or “sink” land-use types are interrelated could be explored in future studies [23].

Our research showed that cultivated land, orchards, construction land, and unused land were positively correlated with each water quality indicator. The correlation coefficients of orchards were lower than those of most coefficients for cultivated land, which could be due to the higher application of fertilizers and pesticides in cultivated land than in orchards. Furthermore, the looser soil texture and lower amount of vegetation in cultivated land than in orchards can enhance the transition of soil nutrients [28]. Previous studies have shown that cultivated land and orchards have the highest correlation with the water pollution index [10]. This indicates that agricultural pollution is currently an important source of non-point source pollution.

The transmission of nutrients from soil to rivers is a process during which the concentration of nutrients before they reach water bodies can be controlled by adjusting the combination of different landscape types in a given spatial pattern, thus reducing the risk of NPS pollution [17]. The landscape pattern index at the landscape level in each sub-basin exhibited some correlation with nutrient indicators in the basin [42]. In this study, patch density (PD), which reflects the degree of landscape fragmentation, exhibited a significant positive correlation with TN. The measurements of COD and electric conductivity indicated that the more complex the landscape type, the better the function of the fixation and retention of nutrients in the water body. LPI characterizes landscape dominance, and LSI shows the landscape shape complexity; the latter had a positive correlation with all nutrient indicators, among which TP and electric conductivity showed a highly significant positive correlation, indicating that the lower the complexity of the landscape composition, the lower the influence on nutrient concentration in the water body. The fractal dimension index area-weighted mean (FRAC-AM) had little correlation with water quality indicators in general, while CONTAG was negatively correlated with all water quality indicators. The greater the CONTAG value, the better the water quality of the river, indicating that there are dominant patches of aggregation and connectivity in the landscape; that is, good aggregation and connectivity of woodland lead to pollution retention, which shows a highly significant negative correlation with TN and electric conductivity. This indicates that woodland has a better TN retention capacity than other land-use types. Furthermore, SHDI was positively correlated with all five water quality indicators, including highly significant positive correlations with TN and TP, and significant positive correlations with conductivity, indicating that the dominant role of woodland as a “sink” landscape decreases with an increase in landscape heterogeneity [22].

A comparative landscape spatial load index (LCI) greater than 0 indicates that the pollution output of the “source” landscape exceeds the pollution retention of the “sink” landscape and that there is a risk of non-point source pollution within the sub-basin; the larger the LCI value, the higher the output risk. The opposite is true if the “sink” landscape is dominant and the risk of non-point source pollution is low [8,43]. The value of the global Moran index of *LWLCI* was 0.701 (*p* < 0.01), indicating that the *LWLCI* in the study area and its spatial distribution were intrinsically, positively linked. As can be seen from the results, there was a significant, positive correlation between *LWLCI* and the water quality indicators of the basin. TN, COD, and conductivity were highly significantly correlated with the *LWLCI* in both study periods. The correlation between *LWLCI* and TN was greatest in September at 0.729, and exceeded that in May, which was consistent with the change in NH_4_^+^-N and was related to the application of large amounts of N fertilizers during this time period, as well as the transmission of N elements from the soil into the water with the increase in summer precipitation. The correlation between COD in the two time periods and that of *LWLCI* in the two time periods was not very different. Both exhibited highly significant correlations, indicating that each small trend of COD in the two time periods was more consistent. However, TP and electric conductivity were more correlated with *LWLCI* during May than in September, and the correlation coefficients decreased over time, indicating that the *LWLCI* in May reflected the changes in TP and conductivity somewhat better than that in September.

The distribution of NPS pollution in the different watersheds of Qixia varied between May and September. According to the correlation between the distribution of land-use types and water quality indicators during May and September, surface runoff and soil erosion caused by precipitation played a role in surface pollution. The fertilization of agricultural land is the main cause of N eutrophication and the decrease in water quality. The usage of land for construction, industrial, and domestic water-use purposes in towns and cities is a source of the total P pollution in rivers. Additionally, the COD and EC indicators in Fengping, where watersheds contain a high proportion of orchards, were found to exceed more than those in other regions, which was likely related to the application of organic fertilizers. Therefore, effective control of all types of land-use practices can be used to control and decrease surface source pollution.

The landscape spatial load comparison index constructed in this study did not consider other factors affecting the formation of non-point source pollution, such as rainfall and soil, and our evaluation model is more suitable for areas with similar soil and rainfall conditions. When studying areas with large differences in rainfall conditions and soil properties, researchers should consider landscape patterns and ecological processes. Corresponding technical treatments, such as appropriate weight assignment for spatial variability in rainfall and soil, should be conducted. Moreover, the samples used in this study were collected within one year. In future studies, the dispersion and accumulation processes of surface pollution caused by changes in landscape patterns over long periods of time should be explored.

Local authorities should pay more attention and actively respond to the water pollution problem in Qixia County. Many studies have already discussed the mitigation of agricultural NPS pollution. Relevant measures include increasing the application of organic manure fertilizers and improving the awareness of local farmers of the severity of water pollution. Authorities should also establish standards for the application of agricultural fertilizers and pesticides based on local agricultural practices to improve their utilization rate [12].

## 5. Conclusions

In this study, the weighted contrast index was constructed based on the “source-sink” landscape theory, and the correlation between landscape patterns and water quality was analyzed. Considering the factors affecting non-point source pollution, the risk of non-point source pollution in a mountainous river basin was assessed. The main conclusions were as follows:

(1) The water quality indicators of the six main rivers in Qixia County varied across different periods. The TN indicator was the highest, greatly exceeding the surface water V standard; the concentration of pollutants in different sections of the same river showed some temporal and spatial differences. Based on the comprehensive pollution index, the water quality of Qixia County was found to generally be good, and most of the water body belonged to the third category or higher. The water quality of the main stream and its tributaries was not significantly different between May and September. The average values of various water quality indicators of the main stream were higher than those of the tributaries during May but lower during September.
(2) The proportion of each land-use type in the 21 sub-basins of Qixia County was moderately different. The proportion of woodland areas was 38.43%, and these areas covered the highest proportion of land in Qixia County. The proportions of different land-use areas had certain correlations with the water quality indicators. Cultivated land and orchards were positively correlated with NPS pollution indicators, while forest land was negatively correlated with water quality indicators. Cultivated land, orchards, construction land, and unused land were the “source” landscapes in the basin. The areas of woodlands, grasslands, and water were negatively correlated with the quality of nutrient salts and were the main “sink” landscapes in the basin.
(3) The spatial load contrast index of the “source” and “sink” landscapes showed a significant spatial correlation in the region and had a significant positive correlation with all NPS pollution indicators in the basin. TN, COD, and electric conductivity were significantly correlated during both periods and can be used as indicators of non-point source pollution.
(4) The heavily polluted area was located in the hilly comprehensive grain-fruit utilization area in the southwest, covering one-seventh of Qixia County. The potential pollution areas were located in the low mountain forest protection areas of Tingkou Town, Miaohou Town, and Taocun Town. The moderately polluted district area accounted for 80% of the total area of Qixia County, indicating that the NPS pollution situation of Qixia County is generally light. Future mitigation measures that comply with local topographic features should be considered in high-*LWLCI* regions.
(5) Whether the high incidence of liver cancer in the Yantai City region, where Qixia is located, is related to the dietary habits or water quality conditions in the region requires further exploration. The large quantities of fertilizers and pesticides applied in Qixia have greatly affected water quality and the local environment. The high level of nitrogen-related contaminants in the water quality of Qixia also requires the attention of the local authorities. This study provides a scientific basis for further potential measures from the relevant sectors.

## Figures and Tables

**Table 1:** Water quality of Qixia in June 2020 (mg/L).

pH	Al	Fe	Mn	Cu	Zn	COD	NO_3_^−^	CL^−^
8.17	0.008	<0.01	<0.008	<0.2	<0.01	2.37	0.9	54.6

**Table 2:** Comparison of fertilizer and pesticide usage in Qixia County, Shandong Province, and China.

	Qixia County	Shandong Province (2018)	China (2018)
Total sown area (km^2^)	1075	110,768	1,659,024
Total fertilizer application (t)	126,501	4,203,500	56,530,000
Average fertilizer application (t/km^2^)	117.67	37.94	34.07
Total pesticide application (t)	4470	130,000	1,503,600
Average pesticide application (t/km^2^)	4.10	1.17	0.91

**Table 3:** Area and percentage of each “source” and “sink” land-use type.

		Area (km^2^)	Percentage
Source landscapes	Construction	147.61	12.52%
	Farmland	395.34	33.53%
	Orchard	636.15	53.95%
	Total area of source landscapes	1179.1	100
Sink landscapes	Water body	21.66	2.93%
	Forests	706.24	95.42%
	Grassland	12.25	1.66%
	Total area of sink landscapes	740.15	100

**Table 4:** Weight of each “source” and “sink” land use type.

“Source”	Weight	“Sink”	Weight
Construction land	0.8	Forests	0.8
Orchards	0.6	Grasslands	0.5
Farmlands	0.6	Water bodies	0.4
Unused land	0.2	-	-

**Table 5:** Water quality test results in Qixia.

Pollution Index (P)	Percentage	Quality Level	Indication
P < 2.0	22.80%	I–II	Very good
2.0 < P < 4.0	71.93%	III	Good
4.0 < P < 8.0	5.27%	IV	Average
8.0 < P < 12.0	0	V	Poor
P > 12.0	0	VI	Worst

**Table 6:** *LWLCI* classification and percentage of area covered.

Score	Classification	Percentage
<0.3	Low	6.86
0.3–0.4	Moderately low	31.33
0.4–0.5	Moderate	19.51
0.5–0.6	Moderately high	37.47
>0.6	High	4.83

**Table 7:** Correlation between the *LWLCI* and water quality indicators.

		TN	TP	NH_4_^+^-N	COD	Electric Conductivity
May	*LWLCI*	0.628 **	0.751 **	0.521 *	0.736 **	0.674 **
September	*LWLCI*	0.729 **	0.527 *	0.707 **	0.726 **	0.562 **

* θ < 0.05, significance level Sig(θ) (significant correlation); ** θ < 0.01, significance level Sig(θ) (highly significant correlation); TN, total N, NH_4_^+^-N, ammonium N, TP, total P, COD, chemical oxygen demand.

## Data Availability

Data available on request due to restrictions. The data presented in this study are available on request from the corresponding author. The data are not publicly available due to the field survey and lab processing.
